# Serum Creatinine Modifies Associations between Body Mass Index and Mortality and Morbidity in Prevalent Hemodialysis Patients

**DOI:** 10.1371/journal.pone.0150003

**Published:** 2016-03-01

**Authors:** Yukitoshi Sakao, Toshiyuki Ojima, Hideo Yasuda, Seiji Hashimoto, Takeshi Hasegawa, Kunitoshi Iseki, Yoshiharu Tsubakihara, Akihiko Kato

**Affiliations:** 1 Blood Purification Unit, Hamamatsu University School of Medicine, Hamamatsu, Shizuoka, Japan; 2 Department of Community Health and Preventive Medicine, Hamamatsu University School of Medicine, Hamamatsu, Shizuoka, Japan; 3 Internal Medicine I, Hamamatsu University School of Medicine, Hamamatsu, Shizuoka, Japan; 4 Committee of Renal Data Registry, Japanese Society for Dialysis Therapy, Tokyo, Japan; Children's National Medical Center, Washington, UNITED STATES

## Abstract

**Background:**

High body mass index (BMI) is paradoxically associated with better outcomes in hemodialysis (HD) patients. This study aimed to examine whether serum creatinine (Cr), a marker of muscle mass, could modify the association between BMI, and mortality and morbidity in prevalent HD patients.

**Methods:**

A retrospective study was conducted using a nationwide database from the registry of the Japanese Society for Dialysis Therapy. A total of 119,099 patients were selected (age: 65±12 years; median time on HD: 5.6 years; male: 62%), and we examined the association of basal BMI with mortality and morbidity after a 1-year period. Patients were stratified either by BMI into 4 groups or by serum Cr levels into 3 tertiles. Odds ratio (OR) [95% confidence interval] was calculated by multivariate logistic regression analysis.

**Results:**

Higher BMI did not predict a higher 1-year total mortality. However, when we stratified the patients by serum Cr levels, the risk of cardiac death became significantly higher in obese patients with the lowest Cr levels, in both males (OR 2.82 [1.51–5.27], p<0.01) and females (OR 2.00 [1.03–3.90], p<0.05). The risk of new cerebral infarction was also higher in obese male patients within the lowest Cr tertile. In contrast, there was a significantly lower risk of cardiac, cerebrovascular, and infection-related death in non-obese patients with higher levels of Cr. Higher serum Cr was also related to a lower risk of cardiovascular events and hip fracture in non-obese HD patients.

**Conclusions:**

The obesity paradox was found to be present in HD patients only when obesity was defined by BMI. Decreased serum Cr levels were found to be positively associated with clinical poor outcomes in all BMI groups. Thus, irrespective of BMI, the evaluation of serum Cr levels is important to predict mortality and morbidity in patients receiving regular HD.

## Introduction

Body mass index (BMI) is a simple and useful marker in the assessment of body size. In general, the mortality risk is lowest in subjects with 22.5–25.0 kg/m^2^ BMI. Higher BMI increases the risk of cardiovascular mortality, while infection- and cancer-related mortality increases at a lower BMI [1]. In contrast, obesity (BMI≥30 kg/m^2^) provides better prognosis in patients with chronic kidney disease (CKD). This association, called “*reverse epidemiology*” [2] or “*obesity paradox*” [3], has been widely observed in different geographic regions and races [4–8].

A recent cohort study showed that mortality risk associated to BMI was comparable between the hemodialysis (HD) and general population, when age and follow-up periods were identical [9]. It has also been reported that obesity is a strong risk factor in HD patients younger than 65 years [10]. Thus, the relationship between BMI and mortality is still controversial, and may underlie complex implications between both associations [11, 12]. BMI becomes a mortality risk factor when whole body composition is considered [13], or in the presence of protein-energy wasting (PEW) [14].

Sarcopenia, defined as decreased skeletal muscle mass and quality, could lead to not only physical inactivity but also poor prognosis in the general elderly [15], and is found to be more prevalent in dialysis patients (37.0% in men and 29.3% in women) [16]. In addition, in HD patients, visceral fat accumulates irrespective of their BMI [17]. Thus, the characteristics of body composition are skeletal muscle atrophy with abdominal adiposity (obese sarcopenia). However, since BMI does not accurately differentiate between lean and fat tissues, a simple measurement of BMI is difficult to depict the independent prognostic effect of each individual tissue.

The aim of this study is to further understand potential factors underlying the association between BMI and clinical outcomes in the dialysis population. We focused on serum creatinine (Cr) (a marker of muscle mass volume) in patients with end-stage kidney disease, and examined whether serum Cr level modifies the correlation of BMI with clinical outcomes in prevalent HD patients.

## Materials and Methods

### Data source

The Japanese Society for Dialysis Therapy (JSDT) has been conducting annual questionnaire surveys for all dialysis facilities since 1968. Data are collected by year-end survey questionnaires sent to dialysis facilities each year, requesting information on each patient. Using this nationwide cohort, we conducted a retrospective analyses of the database collected both at the end of 2008 and 2009 [18, 19]. The study was approved by Institutional Review Board of JSDT and conducted in accordance with the Declaration of Helsinki. Either written or oral informed consent was obtained according to the policy of each dialysis facility. Patient records/information was anonymized and de-identified prior to analysis.

### Patient selection

**Fig 1:**
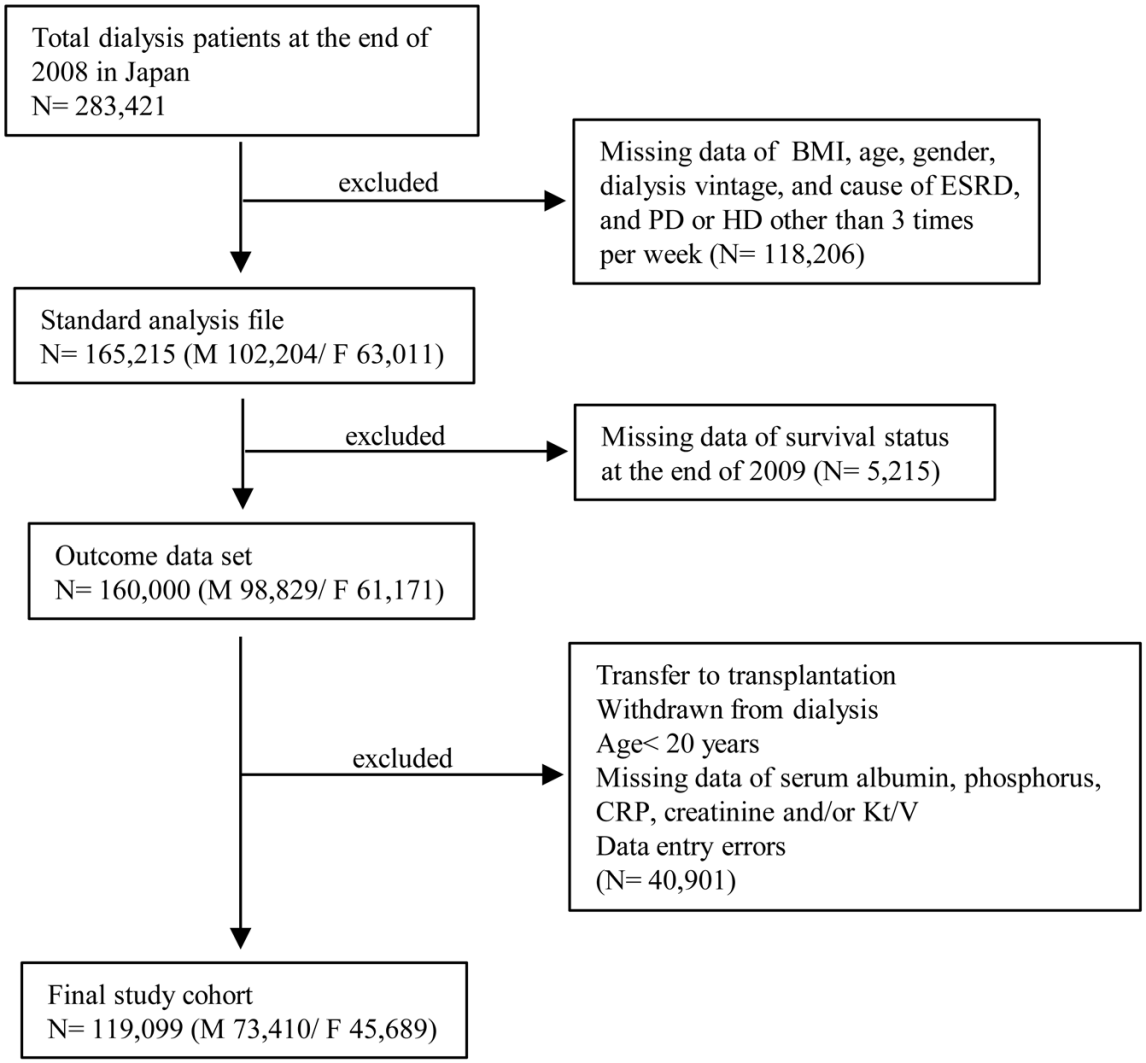
Patient selection. Fig 1 represented how to select and enroll the patients into analyses. Abbreviations are BMI: body mass index, ESRD: end-stage renal disease, PD: peritoneal dialysis, HD: hemodialysis and CRP: C-reactive protein.

The method of patient selection is shown in Fig 1. The 2008 JSDT registry had 283,421 dialysis patients, 273,237 of whom had gender information [18]. We selected 165,215 patients after excluding those treated with peritoneal dialysis, HD therapy other than thrice a week, and those without any record for height or body weight. Of these, 160,000 patients had records regarding their survival status at the end of 2009.

We further excluded patients younger than 20 years of age, those who had stopped HD during the study period, those who had received a kidney transplant, and those whose clinical parameters had not been obtained (Fig 1).

### BMI categories

BMI (kg/m^2^) was calculated by dividing post-HD body weight (kg) by squared body height (m). Based on the World Health Organization (WHO) criteria [20], patients were classified into eight groups. We also categorized BMI less than 18.5 kg/m^2^ as underweight, BMI from 18.5 to 24.9 kg/m^2^ as normal weight [reference], BMI from 25.0 to 29.9 kg/m^2^ as overweight, and BMI more than 30.0 kg/m^2^ as obese.

These groups were further subdivided into 3 tertiles based on serum Cr levels (lowest, middle, and highest tertiles, respectively):

Males: <9.8, 9.8–12.3 [reference], ≥12.4 mg/dl

Females: <8.4, 8.4–10.3 [reference], ≥10.4 mg/dl)

### Clinical outcomes

Causes of death were categorized by JSDT using the 10^th^ edition of the International Classification of Diseases (ICD-10) codes. We recorded the causes of cardiovascular death as cardiac death (cardiac failure due to pulmonary edema, ischemic heart disease, arrhythmia, valvular heart disease, endocarditis, cardiomyopathy, pericarditis, cardiomyositis, pulmonary embolism, or sudden death) or cerebrovascular (CV) death (including cerebral infarction, intracranial hemorrhage, subarachnoid hemorrhage, or other CV diseases).

We estimated the onset of non-fatal myocardial infarction, cerebral infarction, cerebral bleeding, limb amputation, and hip fracture during the period by comparing the records of past history between the 2008 and 2009 datasets. If the disease had been initially recorded in the 2009 file, we judged this as a new event during the 1-year period. The rates of myocardial infarction, cerebral infarction, and cerebral bleeding, combined with fatal and non-fatal events, were then calculated.

### Statistical analyses

Data were expressed as mean ± standard deviation (SD) or median [interquartile range]. Continuous variables were compared using the Jonckheere-Terpstra trend test. Chi-square test was used to compare categorical variables.

Odds ratio (OR [95% confidence intervals (CI)]) was calculated by logistic regression analysis using unadjusted and multivariate adjusted models in each sex. Model 1 was adjusted for age. Model 2 was adjusted for variables in model 1, plus time on HD and presence of diabetes mellitus (DM) as a primary cause of end-stage renal disease (ESRD). Model 3 was adjusted for variables in model 2, plus serum Cr, albumin, phosphorous, C-reactive protein (CRP) and dialysis dose (Kt/V_urea_) assessed by the single-pooled urea kinetic model. Percentile creatinine generation rate (%CGR) [21] and normalized protein catabolic rate (nPCR) [22] were also calculated from Shinzato’s formula.

All calculations were performed using SPSS statistics 17.0 for Windows (SPSS Inc., CA, USA).

## Results

### Patient characteristics

The final number of the enrolled patients was 119,099 (Fig 1). Mean age was 65±12 years in males, and 66±13 years in females. Median time on HD was 5.3 years in men and 6.2 years in women (Table 1, S1 Table). The prevalence of DM was 37.5% in males and 29.2% in females.

**Table 1:** Baseline characteristics of the study cohort.

		Underweight			Normal weight			Overweight			Obesity		
		BMI< 18.5 kg/m^2^			BMI 18.5–24.9 kg/m^2^			BMI 25.0–29.9 kg/m^2^			BMI≥ 30.0 kg/m^2^		
	Total	Lowest Cr	Middle Cr	Highest Cr	Lowest Cr	Middle Cr	Highest Cr	Lowest Cr	Middle Cr	Highest Cr	Lowest Cr	Middle Cr	Highest Cr
Number (%)													
M	73410 (100)	6326 (8.6)	3692 (5.0)	1885 (2.6)	15485 (21.1)	18345 (25.0)	18077 (24.6)	1794 (2.4)	2577 (3.5)	3921 (5.3)	263 (0.4)	320 (0.4)	725 (1.0)
F	45689 (100)	5900 (12.9)	4211 (9.2)	3342 (7.3)	7893 (17.3)	8970 (19.6)	9918 (21.7)	1176 (2.6)	1508 (3.3)	1835 (4.0)	234 (0.5)	285 (0.6)	417 (0.9)
Age (y)													
M	65±12	72±10	66±11	55±14	71±10	66±10	58±11	65±12	63±11	56±11	57±13	54±12	49±10
F	66±13	74±11	67±12	58±12	73±11	68±11	60±11	69±11	67±11	60±11	64±12	62±11	54±12
HD vintage (y)													
M	5.3 [2.3–10.2]	4.3 [1.7–9.0]	8.2 [3.9–14.7]	9.8 [5.3–15.8]	2.6 [0.9–5.8]	5.7 [2.8–10.6]	7.8 [4.4–12.8]	1.8 [0.7–3.5]	3.8 [2.0–6.8]	5.8 [3.3–9.4]	1.9 [0.7–3.8]	3.2 [1.7–5.7]	4.8 [2.7–7.6]
F	6.2 [2.8–11.9]	4.9 [2.0–10.7]	8.7 [4.2–16.3]	10.8 [6.1–17.0]	3.2 [1.3–6.8]	6.3 [3.1–11.7]	8.8 [4.8–14.2]	2.2 [1.0–4.3]	4.5 [2.4–7.7]	6.5 [3.6–10.3]	2.2 [0.8–3.8]	3.7 [2.1–6.1]	5.3 [2.8–8.5]
DM (%)													
M	37.5	39.2	24.8	10.4	51.3	39.8	21.6	65.2	54.9	37.3	70.0	62.2	44.3
F	29.2	31.2	15.5	6.2	46.3	30.9	15.5	66.2	52.9	31.7	74.4	62.5	42.0
P (mg/dL)													
M	5.3±1.4	4.5±1.4	5.3±1.4	5.7±1.4	4.7±1.3	5.3±1.3	5.8±1.4	4.8±1.2	5.5±1.3	6.1±1.5	5.1±1.4	5.8±1.5	6.6±1.6
F	5.3±1.4	4.6±1.4	5.2±1.3	5.6±1.3	4.7±1.3	5.2±1.3	5.7±1.3	4.9±1.1	5.4±1.3	5.9±1.4	5.0±1.3	5.5±1.3	6.2±1.6
Alb (g/dL)													
M	3.7±0.4	3.4±0.5	3.7±0.4	3.9±0.4	3.6±0.5	3.7±0.4	3.9±0.4	3.7±0.4	3.8±0.4	3.9±0.3	3.7±0.4	3.8±0.4	3.9±0.3
F	3.7±0.4	3.4±0.5	3.7±0.4	3.9±0.4	3.5±0.5	3.7±0.4	3.8±0.4	3.6±0.4	3.7±0.3	3.8±0.3	3.6±0.4	3.6±0.3	3.7±0.3
CRP (mg/dL)													
M	0.13 [0.06–0.40]	0.30 [0.10–1.01]	0.12 [0.06–0.40]	0.08 [0.04–0.20]	0.18 [0.07–0.58]	0.13 [0.06–0.37]	0.10 [0.05–0.25]	0.19 [0.09–0.50]	0.19 [0.08–0.45]	0.16 [0.08–0.39]	0.22 [0.10–0.50]	0.30 [0.10–0.74]	0.25 [0.10–0.55]
F	0.10 [0.05–0.30]	0.17 [0.06–0.60]	0.10 [0.04–0.23]	0.05 [0.03–0.11]	0.14 [0.06–0.48]	0.10 [0.05–0.29]	0.09 [0.05–0.20]	0.16 [0.07–0.46]	0.17 [0.07–0.45]	0.13 [0.06–0.36]	0.24 [0.11–0.59]	0.28 [0.10–0.72]	0.27 [0.10–0.71]
Kt/V													
M	1.31 ±0.26	1.37 ±0.30	1.45 ±0.27	1.43 ±0.24	1.25 ±0.28	1.34 ±0.24	1.33 ±0.21	1.11 0.27±	1.22 ±0.23	1.22 ±0.20	1.07 ±0.29	1.13 ±0.25	1.12 ±0.21
F	1.53 ±0.31	1.56 ±0.35	1.67 ±0.31	1.67 ±0.28	1.45 ±0.33	1.56 ±0.29	1.55 ±0.26	1.32 ±0.31	1.41 ±0.28	1.41 ±0.24	1.21 ±0.34	1.32 ±0.26	1.28 ±0.21

Abbreviation: HD, hemodialysis; DM, diabetes mellitus; Cr, serum creatinine; P, serum phosphorus; Alb, serum albumin; CRP, serum C-reactive protein

As BMI increased, the prevalence of DM as well as levels of serum Cr, phosphorous, albumin, and CRP increased, whereas patient age, dialysis vintage, and Kt/V_urea_ decreased (p<0.001) (Table 1, S1 Table).

### Clinical outcomes during the observation

Of the 119,099 patients, 8,854 (7.4%) patients expired during the 1-year study (male/female = 5,685/3,169). The overall incidence of all-cause mortality was 7.73 (male/female = 8.07/7.20) per 100 person-years. The main causes of death were cardiac (32.8%), infection (19.1%), cancer (10.3%), and CV involvement (9.6%).

The incident rates of fatal and nonfatal events recorded per 100 person-years were as follows: myocardial infarction, 2.58 (male/female = 2.92/2.06); cerebral bleeding, 1.75 (male/female = 1.85/1.58); cerebral infarction, 4.48 (male/female = 4.64/4.22); limb amputation, 0.86 (male/female = 0.98/0.68); and hip fracture, 1.16 (male/female = 0.76/1.81).

### Association of BMI with all-cause and cause-specific mortality

**Fig 2:**
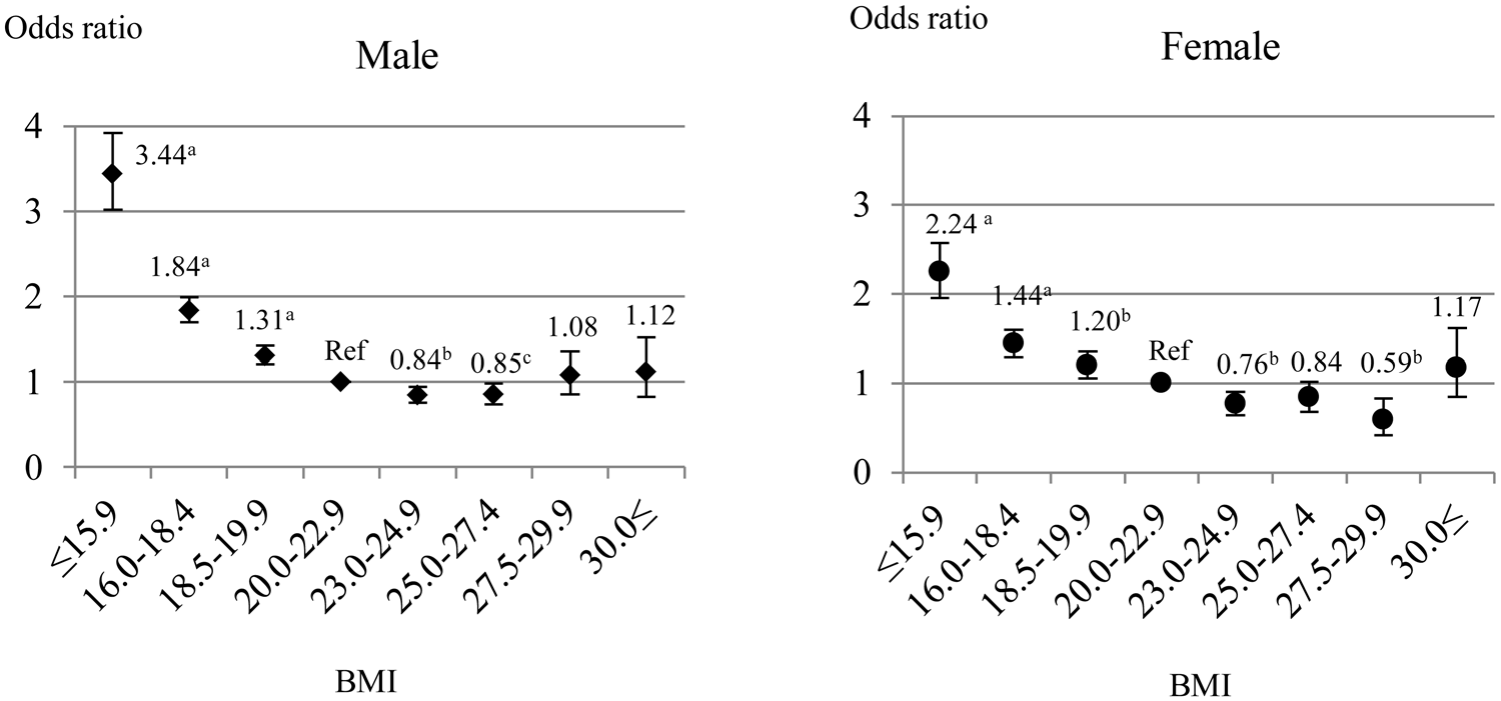
Multivariate-adjusted odds ratios for all-cause mortality according to BMI categories. Odds ratios were calculated by logistic regression analysis adjusted for age, dialysis vintage, diabetes mellitus, serum albumin, phosphorus, CRP, creatinine and Kt/V. We used BMI of 20.0–22.9 kg/m^2^ as the reference. Error bars indicated 95% confidence intervals. Abbreviations are OR: odds ratio, BMI: body mass index, CRP: C-reactive protein and Ref: reference group. ^a^p, ^b^p and ^c^p denoted p values less than 0.001, 0.01 and 0.05 respectively.

The relationship between BMI and all-cause mortality turned out to be an L-shaped curve, after adjusting for multiple covariates (Fig 2). Men exhibited the lowest risk of death at 23.0–24.9 kg/m^2^ BMI (OR 0.84 [95% CI: 0.76–0.94], p<0.01) and women at 27.5–29.9 kg/m^2^ BMI (OR 0.59 [95% CI: 0.42–0.83], p<0.01). A higher BMI (≥30.0 kg/m^2^) did not relate to mortality risk in both genders.

**Fig 3:**
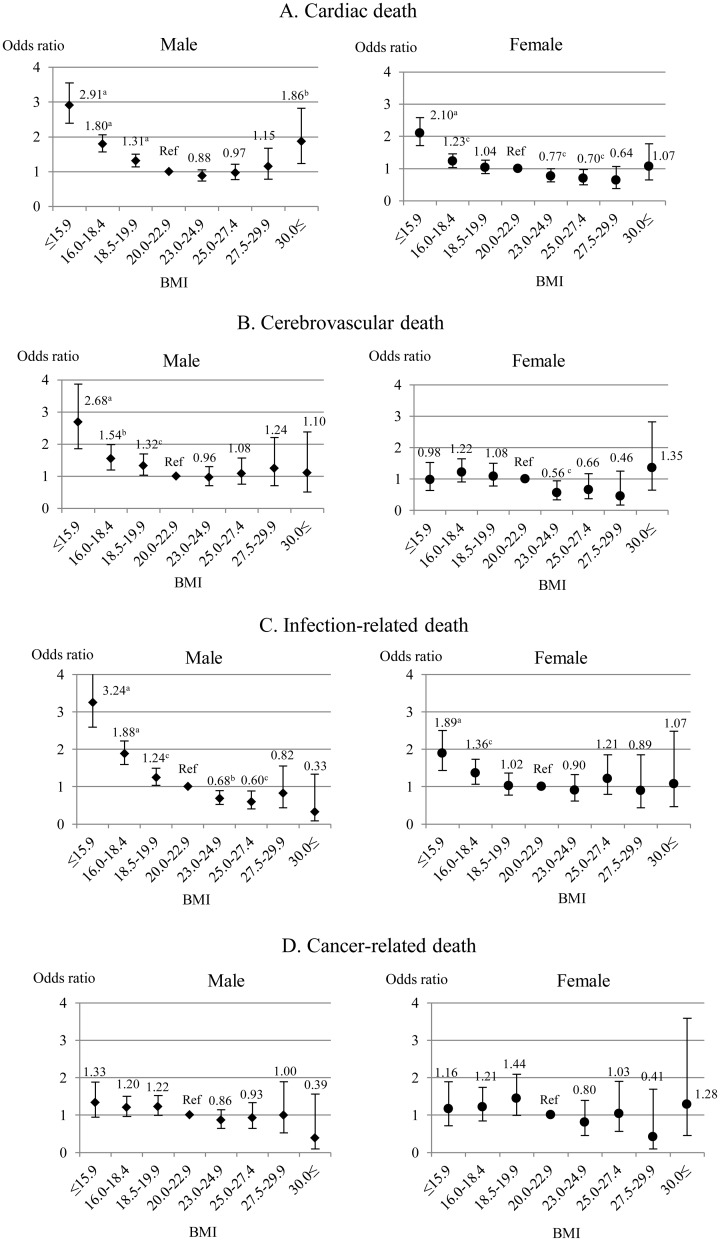
Multivariate-adjusted odds ratios for the cause-specific mortality (A: cardiac death, B: cerebrovascular death, C: infection-related death, D: cancer-related death) according to BMI groups. We used BMI of 20.0–22.9 kg/m^2^ as the reference group. Error bars indicated 95% confidence intervals. Abbreviations are OR: odds ratio, BMI: body mass index and Ref: reference group. ^a^p, ^b^p and ^c^p denoted p values less than 0.001, 0.01 and 0.05 respectively.

There was a significantly higher risk of cardiac, CV- and infection-related mortalities in males with BMI less than 20.0 kg/m^2^ (Fig 3A–3C). The risk of cardiac death was also significantly higher in obese male patients (≥30.0 kg/m^2^). In females, a higher risk of cardiac and infection-related mortality was found in lean patients (BMI<18.5 kg/m^2^). However, there was no connection between cancer-related mortality and BMI categories in both sexes (Fig 3D).

### Relationship between BMI groups and the tertiles of serum Cr

#### 1) All-cause mortality

**Fig 4:**
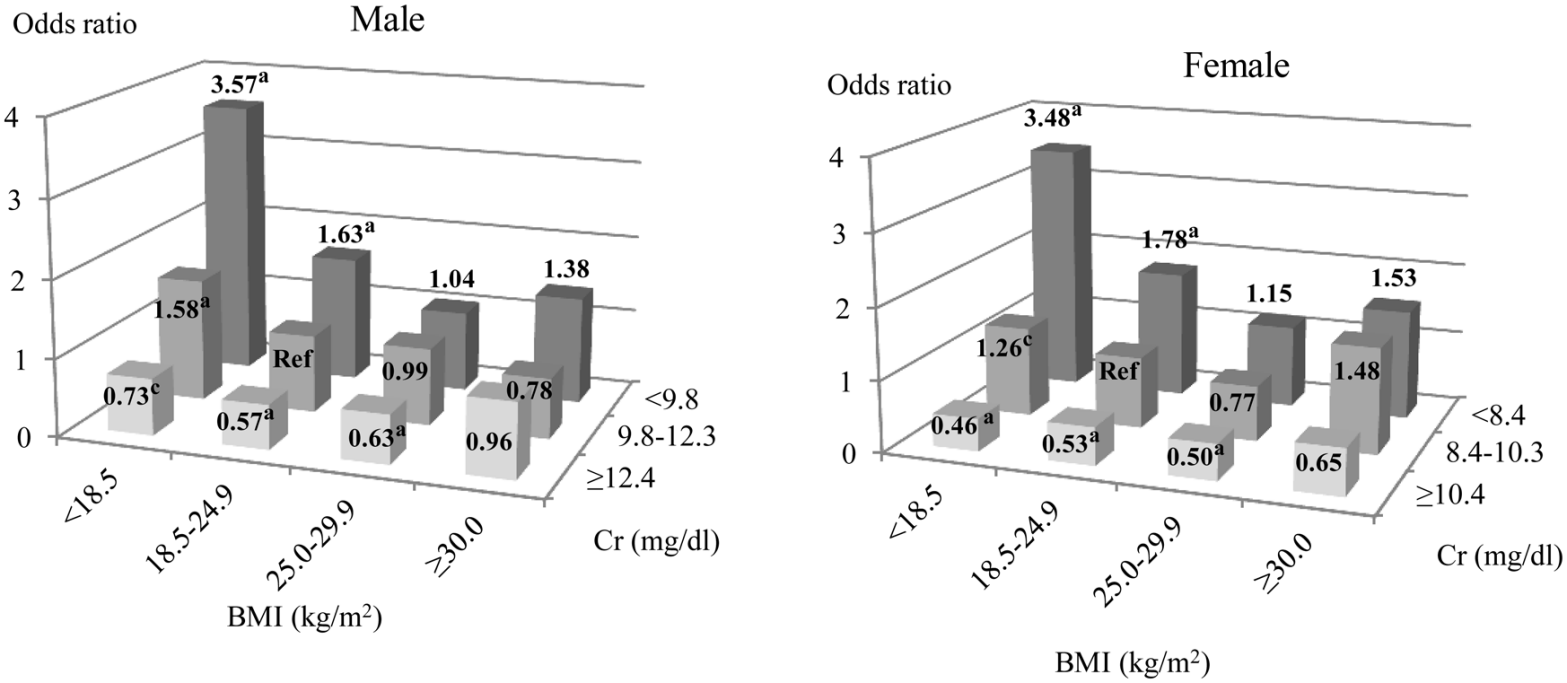
Multivariate-adjusted odds ratios for all-cause mortality according to the BMI groups and the tertiles of serum Cr. We applied the group with normal-weight and the middle tertile of Cr as the reference. Abbreviations are BMI: body mass index, Cr: creatinine and Ref: reference group. ^a^p, ^b^p and ^c^p denoted p values less than 0.001, 0.01 and 0.05 respectively.

In both sexes, there was a significantly higher risk of all-cause mortality in underweight and normal patients within the lowest tertile of Cr. In contrast, all patients within the highest Cr tertile had a lower risk (Fig 4, S2 Table).

The same trend between BMI and serum Cr groups was observed when serum Cr was categorized into quartiles (S1 Fig). There was also an identical trend between BMI and %CGR (S2 Fig). In females, the risk of all-cause death was significantly higher in obese patients in the lowest tertile of %CGR: (OR 1.72 [95% CI: 1.11–2.66], p<0.05) (S2 Fig). In addition, increased serum Cr level was positively associated with higher nPCR (S3 Fig).

#### 2) Cause-specific mortality

**Fig 5:**
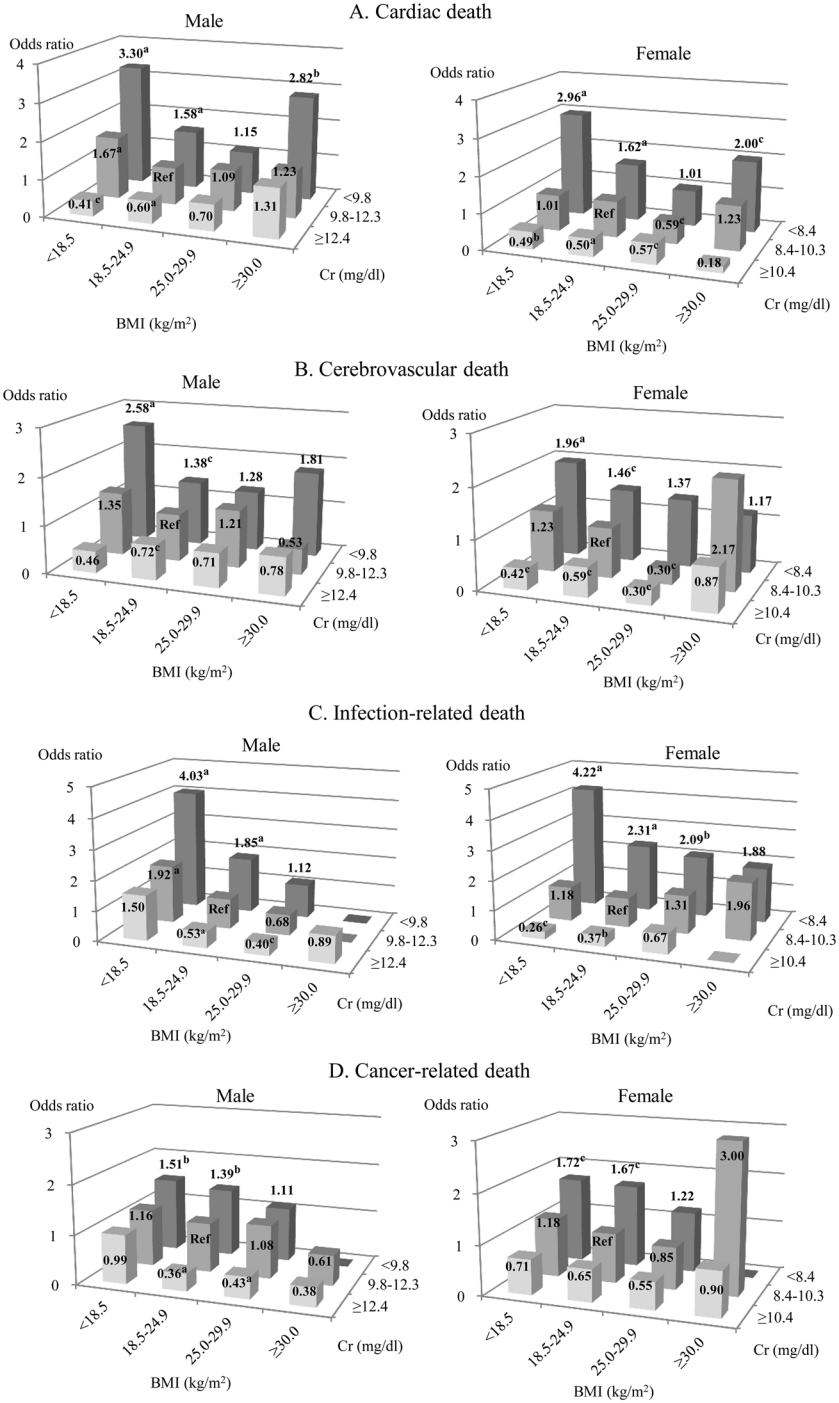
Multivariate-adjusted odds ratios for the cause-specific mortality (A: cardiac death, B: cerebrovascular death, C: infection-related death, D: cancer-related death) according to BMI groups with serum Cr tertiles. We used the mortality rate of the group with normal-weight and the middle tertile of Cr as the reference. Abbreviations are BMI: body mass index, Cr: creatinine and Ref: reference group. ^a^p, ^b^p and ^c^p denoted p values less than 0.001, 0.01 and 0.05 respectively.

In both men and women, the risk of cardiac death was significantly higher in obese patients in the lowest tertile of serum Cr: (OR 2.82 [95% CI: 1.51–5.27], p<0.01) and (OR 2.00 [95% CI: 1.03–3.90], p<0.05) respectively (Fig 5A, S3 Table). In contrast, a higher serum Cr level was significantly related to a lower risk of cardiac death, when BMI was less than 25.0 kg/m^2^ in men and less than 30.0 kg/m^2^ in women.

In both genders, a higher risk of CV and infection- and cancer-related mortality was observed in underweight and normal patients, when serum Cr was in the bottom tertile (Fig 5B–5D, S4–S6 Tables). In contrast, elevated serum Cr (≥12.4 mg/dL) was significantly associated with a lower risk of the above deaths in males. The risk of CV and infection-related mortality was also significantly lower in females with the highest Cr tertile (≥10.4 mg/dL). In obese patients, there was no association between serum Cr and mortality risks.

#### 3) Comorbidity

In both genders, the risk of fatal and nonfatal myocardial infarction was significantly lower in normal patients in the highest Cr tertile, when compared with those categorized into the middle Cr tertile (Tables 2 and 3). The risks of cerebral bleeding, cerebral infarction, limb amputation, and hip fracture were significantly higher in underweight patients whose serum Cr was in the lowest tertile compared to the reference category (Tables 2 and 3). In contrast, there was a significantly lower risk of cerebral infarction in lean patients whose serum Cr was distributed in the top tertile in both male and female population. An increased serum Cr was also related to a lower risk of cerebral bleeding, cerebral infarction, limb amputation, and hip fracture in normal patients. The risks of cerebral bleeding and hip fracture were found to be significantly lower in overweight patients with the highest Cr tertile.

**Table 2:** Associations of BMI with morbidities according to Cr levels in male.

		Categories of BMI (kg/m^2^)			
	Tertile of sCr	<18.5	18.5–24.9	25.0–29.9	≥30.0
**Myocardial infarction**	**Lowest**	1.05 (0.86–1.28)	1.06 (0.92–1.22)	1.18 (0.89–1.58)	1.56 (0.79–3.08)
	**Middle**	0.98 (0.77–1.25)	Reference	0.89 (0.68–1.16)	1.33 (0.70–2.54)
	**Highest**	0.66 (0.43–1.01)	0.74 (0.63–0.86) ^a^	0.79 (0.61–1.03)	0.99 (0.58–1.69)
**Cerebral bleeding**	**Lowest**	1.44 (1.16–1.79) ^b^	1.08 (0.91–1.29)	0.97 (0.66–1.44)	0.51 (0.13–2.08)
	**Middle**	0.97 (0.72–1.30)	Reference	0.71 (0.49–1.04)	0.99 (0.40–2.42)
	**Highest**	0.53 (0.31–0.92) ^c^	0.72 (0.60–0.87) ^b^	0.69 (0.50–0.97) ^c^	0.48 (0.19–1.17)
**Cerebral infarction**	**Lowest**	1.43 (1.24–1.67) ^a^	1.11 (0.99–1.24)	1.00 (0.77–1.29)	1.80 (1.05–3.09) ^c^
	**Middle**	1.15 (0.95–1.39)	Reference	0.87 (0.69–1.10)	0.67 (0.31–1.43)
	**Highest**	0.66 (0.45–0.95) ^c^	0.76 (0.67–0.87) ^a^	0.83 (0.66–1.04)	0.56 (0.30–1.05)
**Limb amputation**	**Lowest**	1.39 (1.04–1.87) ^c^	1.02 (0.81–1.28)	0.91 (0.56–1.47)	1.75 (0.70–4.37)
	**Middle**	0.92 (0.61–1.39)	Reference	0.73 (0.47–1.15)	0.80 (0.25–2.54)
	**Highest**	0.41 (0.17–1.01)	0.45 (0.34–0.61) ^a^	0.72 (0.48–1.09)	0.72 (0.31–1.67)
**Hip fracture**	**Lowest**	2.04 (1.51–2.76) ^a^	1.36 (1.05–1.76) ^c^	0.65 (0.30–1.40)	0.00
	**Middle**	1.29 (0.86–1.95)	Reference	0.67 (0.35–1.28)	1.38 (0.34–5.64)
	**Highest**	0.37 (0.12–1.16)	0.64 (0.46–0.89) ^b^	0.47 (0.23–0.97) ^c^	0.41 (0.06–2.95)

Data are expressed as odds ratio (95% confidence interval) compared to the reference group of BMI 18.5–24.9 with middle tertile of sCr, adjusted for age, dialysis vintage, diabetes mellitus, serum albumin, phosphorus, C-reactive protein and Kt/V.

^a^*P* < 0.001,

^b^*P* < 0.01, and

^c^*P* < 0.05.

Abbreviation: BMI, body mass index; sCr, serum creatinine

**Table 3:** Associations of BMI with morbidities according to Cr levels in female.

		Categories of BMI (kg/m^2^)			
	Tertile of sCr	<18.5	18.5–24.9	25.0–29.9	≥30.0
**Myocardial infarction**	**Lowest**	1.16 (0.91–1.48)	1.00 (0.80–1.25)	0.67 (0.41–1.07)	1.62 (0.81–3.26)
	**Middle**	1.01 (0.76–1.35)	Reference	1.12 (0.78–1.60)	0.76 (0.31–1.88)
	**Highest**	0.74 (0.50–1.10)	0.67 (0.52–0.87) ^b^	0.77 (0.50–1.19)	0.67 (0.27–1.67)
**Cerebral bleeding**	**Lowest**	1.53 (1.17–1.99) ^b^	1.11 (0.86–1.43)	0.48 (0.25–0.93) ^c^	0.70 (0.22–2.23)
	**Middle**	1.06 (0.77–1.46)	Reference	0.33 (0.16–0.67) ^b^	0.63 (0.20–2.01)
	**Highest**	0.84 (0.56–1.25)	0.61 (0.46–0.82) ^b^	0.58 (0.34–0.99) ^c^	0.78 (0.31–1.93)
**Cerebral infarction**	**Lowest**	1.37 (1.15–1.64) ^a^	1.14 (0.97–1.35)	1.21 (0.90–1.63)	1.05 (0.54–2.02)
	**Middle**	0.92 (0.74–1.15)	Reference	1.02 (0.76–1.37)	1.07 (0.57–1.99)
	**Highest**	0.68 (0.50–0.91) ^b^	0.65 (0.53–0.79) ^a^	0.68 (0.48–0.97) ^c^	0.60 (0.28–1.29)
**Limb amputation**	**Lowest**	3.01 (1.96–4.62) ^a^	2.12 (1.41–3.18)^a^	1.37 (0.68–2.73)	1.15 (0.27–4.87)
	**Middle**	1.51 (0.85–2.69)	Reference	0.96 (0.46–2.00)	0.95 (0.23–4.00)
	**Highest**	0.88(0.38–2.01)	0.79 (0.47–1.33)	1.11 (0.54–2.27)	0.71 (0.17–3.00)
**Hip fracture**	**Lowest**	1.89 (1.49–2.39) ^a^	1.24 (0.98–1.57)	0.98 (0.61–1.59)	0.27(0.04–1.98)
	**Middle**	1.07 (0.78–1.46)	Reference	0.81 (0.50–1.32)	0.27 (0.04–1.95)
	**Highest**	0.78 (0.49–1.24)	0.52 (0.37–0.73) ^a^	0.37 (0.17–0.79) ^c^	0.57 (0.14–2.33)

Data are expressed as odds ratio (95% confidence interval) compared to the reference group of BMI 18.5–24.9 with middle tertile of sCr, adjusted for age, dialysis vintage, diabetes mellitus, serum albumin, phosphorus, C-reactive protein and Kt/V.

^a^*P* < 0.001,

^b^*P* < 0.01, and

^c^*P* < 0.05.

Abbreviation: BMI, body mass index; sCr, serum creatinine

## Discussion

Obesity paradox has been well demonstrated in dialysis patients, but is not a universal finding. In a 12-year follow-up, Kaizu et al. [23] observed that survival rate was significantly lower in non-diabetic HD patients with BMI more than 23.0 kg/m^2^ compared to that in those with a BMI of 17.0–18.9 kg/m^2^. During their 2-year observation, Wong et al. [24] also reported that obese Asian-American dialysis patients were associated with poor prognosis. The Netherlands survey also did not find any clinical benefit for obesity [9, 10].

In this study, there was a L-shaped association between BMI and all cause mortality both in men and women. Recently, it is speculated that muscle size can help to explain the complicated relationship between BMI and mortality in chronic illnesses such as cancer [25], heart failure [26], and type-2 DM [27]. Lowrie and Lew first reported a strong relationship between serum Cr and mortality in dialysis patients [28]. In a US nationwide cohort, Kalantar-Zadeh et al. [29] demonstrated that higher BMI with higher serum Cr concentration appears to be associated with a higher survival rate in HD patients. They showed that in HD patients, a decline in serum Cr over time was a stronger predictor of fatality than body weight loss [30]. A trend association between higher Cr and lower risk of total mortality was also observed across all BMI categories in the Dialysis Outcomes Practice Patterns Study (DOPPS) [31].

As expected, our study showed that obesity, as assessed by BMI, was not associated with the risk for all-cause mortality. However, after stratifying obese patients by serum Cr tertiles, the lowest tertile of serum Cr was significantly related to an increased risk of cardiac death and cerebral infarction. In addition, the risk of all-cause or cause-specific death was significantly lower than that in the reference category in lean patients, when serum Cr was classified into the top tertile (Figs 4 and 5). In agreement with the findings, Honda et al. [14] have reported that obese sarcopenia was associated with a higher risk of mortality in incident dialysis patients. Moreau-Gaudry et al also found that serum Cr lower than 8.1 mg/dL was associated with mortality in HD patients with BMI over 23 kg/m^2^ [32]. A recent international cohort study demonstrated that assessment of body lean and fat masses is more useful in predicting survival prognosis in HD patients [33]. Taken together, these observations convincingly suggest that serum Cr level, irrespective of BMI, is more useful than BMI alone in predicting mortality and comorbidity in HD patients.

We showed that the highest tertile of serum Cr was associated with a lower risk of cardiac and CV-related deaths. Increased Cr was also related to a lower risk of fatal and non-fatal cardiovascular events. In general, sarcopenia increases the risk of mortality, independent of BMI category [34]. Thigh sarcopenia is closely associated with arteriosclerotic changes in HD patients [35, 36]. Since serum Cr is positively correlated with nPCR, an indirect marker of dietary protein intake, adequate protein intake may be essential to prevent muscle wasting, thereby leading to a slower progression of arteriosclerosis.

Low BMI is strongly linked to the risk of hip fracture in maintenance HD patients. The risk of hip fracture was 2.5-fold higher in patients with BMI less than 23.0 kg/m^2^ than in those with higher BMI [37]. Sarcopenia has commonly been seen in elderly patients with hip fracture [38]. In this study, we confirmed that the risk of hip fracture was 2.0-fold higher in men and 1.9-fold higher in women with BMI less than 18.5 kg/m^2^ with the lowest tertile of Cr. However, in patients with the highest Cr tertile in this group, the risk of bone fracture became rather low in comparison to the reference. Similarly, elevated Cr levels were also associated with lower risk of hip fracture in patients with normal to excess body weight.

Recently, decreased muscle power in lower extremities has been shown to be strongly associated with increased mortality risk in HD patients [39]. Handgrip strength is also more associated with physical inactivity and mortality than muscle mass [40]. Hence, serum Cr may preferentially reflect muscle function rather than muscle mass volume itself in HD patients.

In the dialysis population, the risk of infection-related death was 7.5-fold higher than in the general population; in patients aged 60–74 years, the risk of infection-related death was as high as 22-fold [41]. Sarcopenia is known to be a potent risk factor for nosocomial infections in the elderly [42]. In this study, a lower serum Cr level was significantly related to a higher risk of infection-related death, when men had BMI less than 25.0 kg/m^2^ and women had BMI less than 30.0 kg/m^2^.

Recently, a low serum Cr level has been demonstrated as an independent risk factor in vancomycin-resistant enterococcal colonization [43] and nosocomial aspiration pneumonia [44] in HD patients. The decrease of serum Cr levels during the first 2 weeks is a risk factor of pneumonia-related mortality [44]. Since swallowing and chewing are closely correlated with systemic loss of skeletal muscle mass volume [38], decreased serum Cr level may be associated with sarcopenic dysphagia, an important cause of recurrent aspiration pneumonia.

We have several limitations to this cohort. Firstly, we did not directly evaluate lean and fat masses by anthropometric measurements. However, since serum Cr correlates well with lean body mass volume [45], measurement of serum Cr is established as a surrogate marker of skeletal muscle mass in HD patients [46]. Secondly, besides muscle mass, pre-dialysis serum Cr level might be affected by residual renal function. In this cohort, the interquartile time on HD was 2.3–10.2 years and 2.8–11.9 years for males and females respectively, indicating that over 75% of the patients had been undergoing HD regularly for more than 2 years. Since residual renal function can generally be considered negligible in patients receiving HD for more than 2 years, serum Cr levels could be a good indicator for muscle mass in patients on long-term HD. Thirdly, we only measured BMI and clinical parameters at commencement due to the methodological limitations in this nationwide cohort. Finally, we did not assess other potentially confounders such as psychological, social, and economic factors.

In summary, our findings indicate that the obesity paradox occurs in HD patients only when obesity is simply defined by BMI. However, when patients are subdivided by serum Cr levels in each BMI category, the obesity paradox disappears. Rather, serum Cr level plays a crucial role in determining mortality and morbidity. Thus, we conclude that an early identification and intervention for sarcopenia (along with obesity) by assessing serum Cr levels may be more important to improve poor clinical outcomes in prevalent HD patients.

## Supporting Information

S1 Fig Multivariate-adjusted odds ratios for all-cause mortality in the four BMI groups and the 4 quartiles of serum Cr. We applied the group with normal-weight and the second quartile of Cr as the reference. Abbreviations are BMI: body mass index, Cr: creatinine and Ref: reference group. ^a^p, ^b^p and ^c^p denoted p values less than 0.001, 0.01 and 0.05 respectively.
(TIF)

S2 Fig Multivariate-adjusted odds ratios for all-cause mortality according to the BMI groups and the tertiles of %CGR. We applied the group with normal-weight and the middle tertile of %CGR as the reference. Abbreviations are BMI: body mass index, %CGR: percentile creatinine gereration rate and Ref: reference group. ^a^p, ^b^p and ^c^p denoted p values less than 0.001, 0.01 and 0.05 respectively.
(TIF)

S3 Fig Normalized protein catabolic rates according to the four BMI categories with serum Cr tertiles. Abbreviations are BMI: body mass index, Cr: creatinine and nPCR: normalized protein catabolic rate. Labeled data indicates mean nPCR in each group.
(TIF)

S1 Table Baseline characteristics of the study cohort. (PDF)

S2 Table Associations of BMI with all-cause mortality according to Cr levels. (PDF)

S3 Table Associations of BMI with cardiac mortality according to Cr levels. (PDF)

S4 Table Associations of BMI with cerebrovascular mortality according to Cr levels. (PDF)

S5 Table Associations of BMI with infection-related mortality according to Cr levels. (PDF)

S6 Table Associations of BMI with cancer mortality according to Cr levels. (PDF)
