# Supplementary figures and images for: Serum Creatinine Modifies Associations between Body Mass Index and Mortality and Morbidity in Prevalent Hemodialysis Patients
Source: PLoS One. 2016 Mar 1;11(3):e0150003. doi: 10.1371/journal.pone.0150003 (PMC4773191; doi:10.1371/journal.pone.0150003)

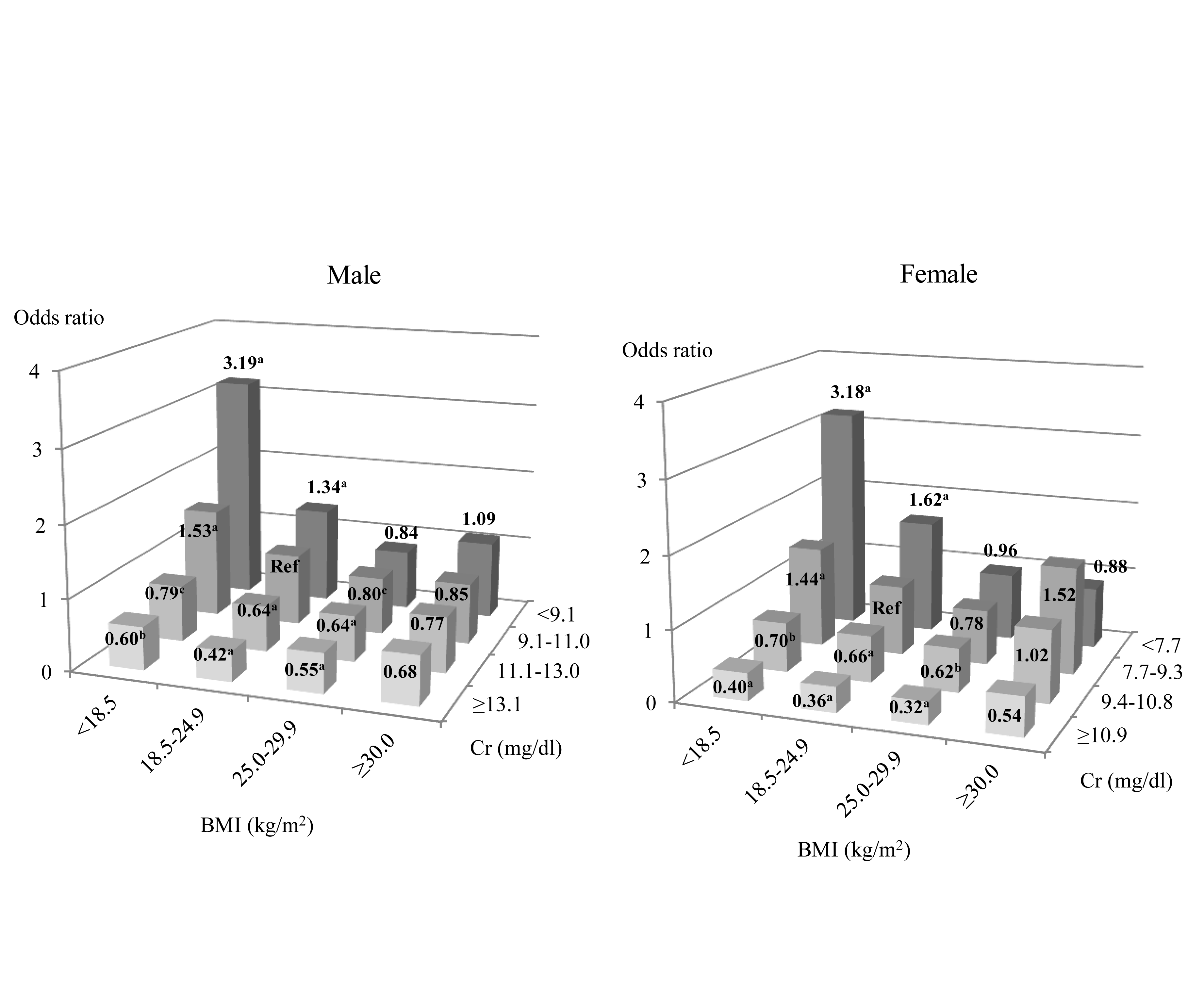

Supplement: S1 Fig — We applied the group with normal-weight and the second quartile of Cr as the reference. Abbreviations are BMI: body mass index, Cr: creatinine and Ref: reference group. ap, bp and cp denoted p values less than 0.001, 0.01 and 0.05 respectively. (TIF) [file pone.0150003.s001.tif]

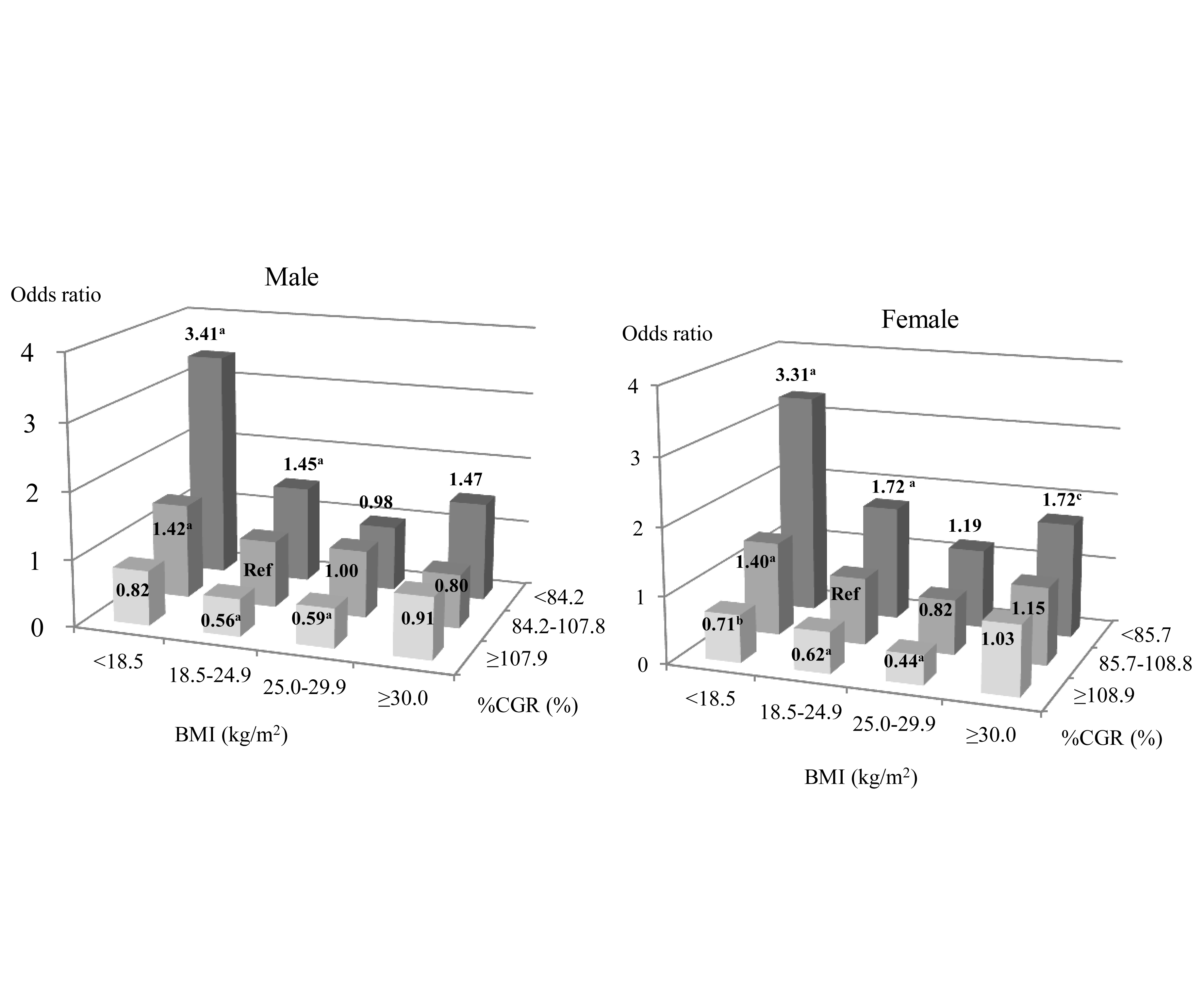

Supplement: S2 Fig — We applied the group with normal-weight and the middle tertile of %CGR as the reference. Abbreviations are BMI: body mass index, %CGR: percentile creatinine gereration rate and Ref: reference group. ap, bp and cp denoted p values less than 0.001, 0.01 and 0.05 respectively. (TIF) [file pone.0150003.s002.tif]

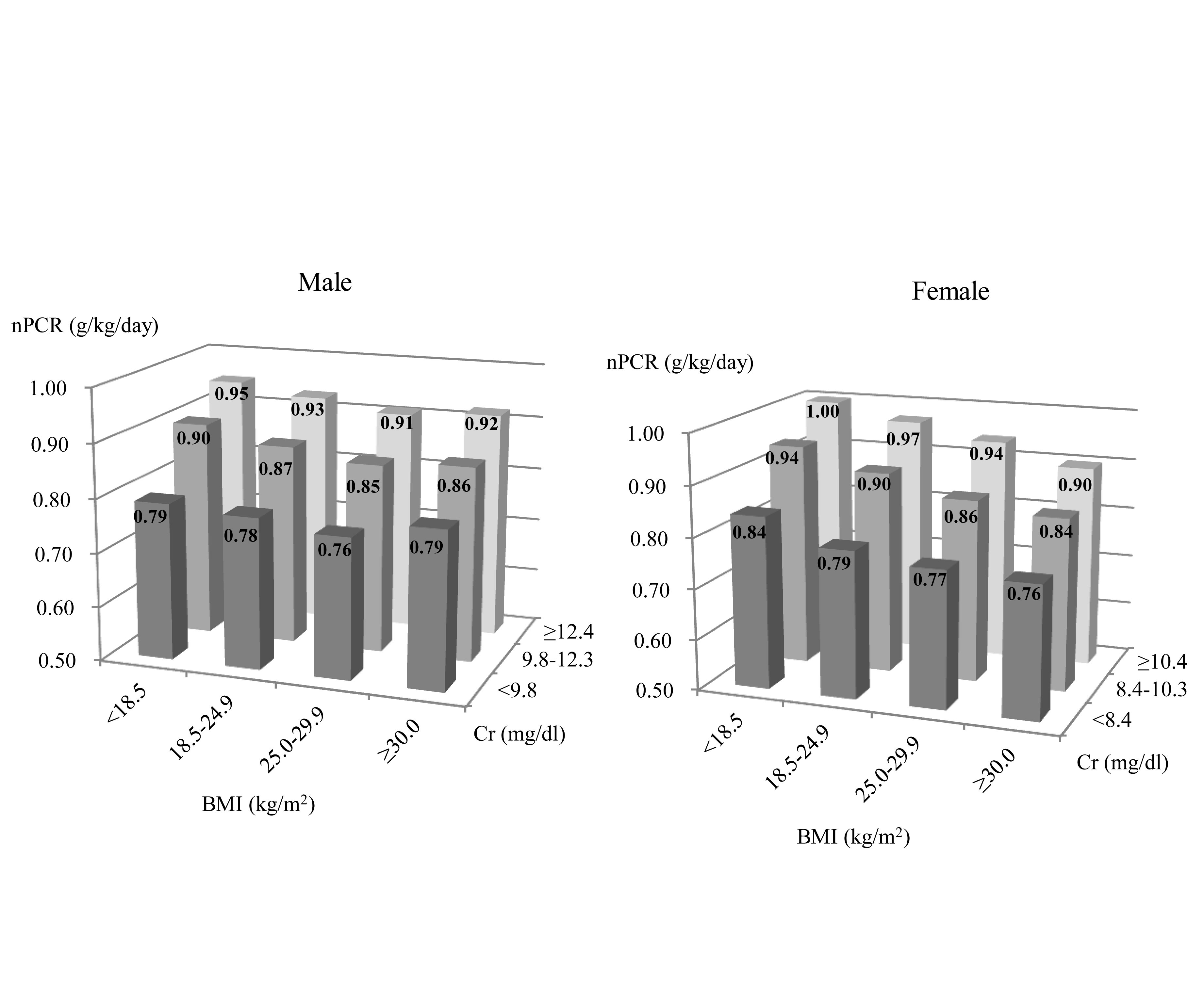

Supplement: S3 Fig — Abbreviations are BMI: body mass index, Cr: creatinine and nPCR: normalized protein catabolic rate. Labeled data indicates mean nPCR in each group. (TIF) [file pone.0150003.s003.tif]
