# Supplementary material for: Serum Creatinine Modifies Associations between Body Mass Index and Mortality and Morbidity in Prevalent Hemodialysis Patients
Source: PLoS One. 2016 Mar 1;11(3):e0150003. doi: 10.1371/journal.pone.0150003 (PMC4773191; doi:10.1371/journal.pone.0150003)
Supplement: S1 Table — (PDF) [file pone.0150003.s004.pdf]

S1 Table. Baseline characteristics of the study cohort

	Total	Categories of BMI (kg/m^2)								p-value
		≤15.9	16.0-18.4	18.5-19.9	20.0-22.9	23.0-24.9	25.0-27.4	27.5-29.9	≥30.0	
**Number (%)**										
**M**	73410	1691	10212	12914	27763	11230	6214	2078	1308	
	(100)	(2.3)	(13.9)	(17.6)	(37.8)	(15.3)	(8.5)	(2.8)	(1.8)	
**F**	45689	2899	10554	8413	13538	4830	3182	1337	936	
	(100)	(6.3)	(23.1)	(18.4)	(29.6)	(10.6)	(7.0)	(2.9)	(2.0)	
**Age (y)**										
**M**	65±12	70±13	67±13	66±12	65±12	63±11	61±12	57±12	52±12	<0.001
**F**	66±13	70±13	67±13	66±13	66±12	66±12	65±12	63±12	59±13	<0.001
**HD vintage (y)**										
**M**	5.3	5.6	6.3	6.0	5.3	4.8	4.1	3.9	3.8	<0.001
	[2.3-10.2]	[2.4-11.1]	[2.7-12.3]	[2.5-12.1]	[2.3-10.3]	[2.2-8.8]	[2.0-7.7]	[1.9-7.3]	[1.9-6.5]	
**F**	6.2	5.6	6.3	6.0	5.3	4.8	4.1	3.9	3.8	<0.001
	[2.8-11.9]	[2.4-11.1]	[2.7-12.3]	[2.5-12.1]	[2.3-10.3]	[2.2-8.8]	[2.0-7.7]	[1.9-7.3]	[1.9-6.5]	
**DM (%)**										
**M**	37.5	29.6	30.2	32.3	36.7	42.7	48.0	51.2	53.8	<0.001
**F**	29.2	18.9	20.4	23.5	30.4	38.9	45.9	52.3	56.3	<0.001
**Cr (mg/dl)**										
**M**	11.1±3.0	8.0±2.8	9.8±2.7	10.6±2.8	11.3±2.8	11.8±2.9	12.0±3.0	12.3±3.2	12.6±3.3	<0.001
**F**	9.3±2.4	7.6±2.4	8.9±2.4	9.4±2.3	9.6±2.3	9.7±2.3	9.8±2.4	9.8±2.5	10.0±2.6	<0.001
**P (mg/dl)**										
**M**	5.3±1.4	4.7±1.6	5.0±1.5	5.2±1.4	5.3±1.4	5.4±1.4	5.6±1.4	5.8±1.5	6.1±1.6	<0.001
**F**	5.2±1.4	4.9±1.5	5.1±1.4	5.2±1.4	5.2±1.4	5.3±1.4	5.4±1.4	5.5±1.4	5.7±1.5	<0.001
**Alb (g/dL)**										
**M**	3.7±0.4	3.3±0.6	3.6±0.5	3.7±0.4	3.7±0.4	3.8±0.4	3.8±0.4	3.8±0.4	3.8±0.4	<0.001
**F**	3.7±0.4	3.5±0.5	3.7±0.5	3.7±0.4	3.7±0.4	3.7±0.4	3.7±0.4	3.7±0.3	3.7±0.4	<0.001
**CRP (mg/dL)**										
**M**	0.13	0.30	0.16	0.11	0.12	0.13	0.17	0.20	0.26	<0.001
	[0.06-0.40]	[0.10-1.18]	[0.06-0.54]	[0.05-0.38]	[0.06-0.35]	[0.07-0.37]	[0.08-0.40]	[0.09-0.49]	[0.10-0.59]	
**F**	0.10	0.12	0.10	0.10	0.10	0.11	0.14	0.18	0.26	<0.001
	[0.05-0.30]	[0.05-0.48]	[0.05-0.30]	[0.05-0.27]	[0.05-0.30]	[0.05-0.31]	[0.06-0.40]	[0.08-0.45]	[0.10-0.68]	
**Kt/V**										
**M**	1.31±0.26	1.42±0.31	1.40±0.27	1.36±0.25	1.31±0.25	1.25±0.23	1.21±0.23	1.17±0.23	1.11±0.24	<0.001
**F**	1.53±0.31	1.64±0.35	1.61±0.32	1.57±0.30	1.52±0.29	1.46±0.29	1.40±0.28	1.36±0.28	1.28±0.27	<0.001

Abbreviation: BMI, body mass index; HD, hemodialysis; DM, diabetes mellitus; Cr, serum creatinine; P, serum phosphorus; Alb, serum albumin; CRP, serum C-reactive protein
