# Supplementary material for: Serum Creatinine Modifies Associations between Body Mass Index and Mortality and Morbidity in Prevalent Hemodialysis Patients
Source: PLoS One. 2016 Mar 1;11(3):e0150003. doi: 10.1371/journal.pone.0150003 (PMC4773191; doi:10.1371/journal.pone.0150003)
Supplement: S2 Table — (PDF) [file pone.0150003.s005.pdf]

S2 Table. Associations of BMI with all-cause mortality according to Cr levels

All-cause death

Male

	Tertile of sCr	Categories of BMI (kg/m²) <18.5	18.5-24.9	25.0-29.9	≥30.0
**Unadjusted**	**Lowest**	5.85 (5.37-6.36) [a]	2.32 (2.14-2.51) [a]	1.20 (0.98-1.46)	1.24 (0.76-2.00)
	**Middle**	1.56 (1.37-1.78) [a]	Reference	0.92 (0.77-1.11)	0.54 (0.29-1.02)
	**Highest**	0.42 (0.31-0.57) [a]	0.38 (0.34-0.43) [a]	0.40 (0.32-0.49) [a]	0.48 (0.31-0.75) [b]
**Model 1**	**Lowest**	4.68 (4.30-5.10) [a]	1.96 (1.80-2.12) [a]	1.22 (1.00-1.48)	1.74 (1.07-2.84) [c]
	**Middle**	1.58 (1.38-1.80) [a]	Reference	1.03 (0.86-1.24)	0.88 (0.46-1.66)
	**Highest**	0.64 (0.47-0.86) [b]	0.52 (0.46-0.59) [a]	0.59 (0.48-0.74) [a]	1.00 (0.63-1.57)
**Model 2**	**Lowest**	4.74 (4.35-5.17) [a]	2.00 (1.85-2.17) [a]	1.27 (1.04-1.54) [c]	1.82 (1.12-2.97) [c]
	**Middle**	1.56 (1.37-1.79) [a]	Reference	1.05 (0.87-1.27)	0.91 (0.48-1.73)
	**Highest**	0.66 (0.49-0.89) [b]	0.54 (0.48-0.61) [a]	0.63 (0.50-0.78) [a]	1.09 (0.69-1.72)
**Model 3**	**Lowest**	3.57 (3.26-3.92) [a]	1.63 (1.49-1.77) [a]	1.04 (0.85-1.28)	1.38 (0.83-2.28)
	**Middle**	1.58 (1.38-1.81) [a]	Reference	0.99 (0.82-1.20)	0.78 (0.41-1.47)
	**Highest**	0.73 (0.54-0.99) [c]	0.57 (0.51-0.65) [a]	0.63 (0.50-0.79) [a]	0.96 (0.61-1.52)

All-cause death

Female

	Tertile of sCr	Categories of BMI (kg/m²) <18.5	18.5-24.9	25.0-29.9	≥30.0
**Unadjusted**	**Lowest**	5.55 (4.93-6.24) [a]	2.72 (2.41-3.07) [a]	1.56 (1.22-2.00) [a]	1.86 (1.15-3.01) [c]
	**Middle**	1.11 (0.94-1.32)	Reference	0.84 (0.64-1.12)	1.42 (0.87-2.32)
	**Highest**	0.26 (0.18-0.35) [a]	0.35 (0.29-0.42) [a]	0.39 (0.27-0.55) [a]	0.47 (0.24-0.91) [c]
**Model 1**	**Lowest**	4.47 (3.97-5.04) [a]	2.28 (2.02-2.58) [a]	1.51 (1.17-1.93) [b]	2.17 (1.34-3.52) [b]
	**Middle**	1.17 (0.98-1.39)	Reference	0.90 (0.68-1.19)	1.82 (1.12-2.98) [c]
	**Highest**	0.39 (0.28-0.54) [a]	0.50 (0.42-0.60) [a]	0.55 (0.38-0.79) [b]	0.80 (0.41-1.57)
**Model 2**	**Lowest**	4.48 (3.97-5.05) [a]	2.32 (2.05-2.63) [a]	1.54 (1.20-1.98) [b]	2.23 (1.37-3.63) [b]
	**Middle**	1.16 (0.98-1.38)	Reference	0.90 (0.68-1.20)	1.87 (1.14-3.07) [c]
	**Highest**	0.40 (0.29-0.55) [a]	0.52 (0.43-0.63) [a]	0.58 (0.40-0.83) [b]	0.86 (0.44-1.68)
**Model 3**	**Lowest**	3.48 (3.07-3.94) [a]	1.78 (1.57-2.03) [a]	1.15 (0.89-1.49)	1.53 (0.93-2.53)
	**Middle**	1.26 (1.06-1.50) [c]	Reference	0.77 (0.58-1.03)	1.48 (0.90-2.43)
	**Highest**	0.46 (0.33-0.64) [a]	0.53 (0.44-0.64) [a]	0.50 (0.35-0.72) [a]	0.65 (0.33-1.28)

Data are expressed as odds ratio (95% confidence interval) compared to the reference group of BMI 18.5-24.9 with middle tertile of sCr.

Model 1: adjusted for age

Model 2: adjusted for age, dialysis vintage, diabetes mellitus

Model 3: adjusted for age, dialysis vintage, diabetes mellitus, serum albumin, phosphorus, C-reactive protein , Kt/V

[a] p<0.001, [b] p<0.01, [c] p<0.05 Abbreviation: BMI, body mass index; sCr, serum creatinine
