# Supplementary material for: Serum Creatinine Modifies Associations between Body Mass Index and Mortality and Morbidity in Prevalent Hemodialysis Patients
Source: PLoS One. 2016 Mar 1;11(3):e0150003. doi: 10.1371/journal.pone.0150003 (PMC4773191; doi:10.1371/journal.pone.0150003)
Supplement: S3 Table — (PDF) [file pone.0150003.s006.pdf]

**S3 Table. Associations of BMI with cardiac mortality according to Cr levels**

**Cardiac death**

**Male**

| | | Categories of BMI (kg/m$^2$) | | | |
|---|---|---|---|---|---|
| | **Tertile of sCr** | **<18.5** | **18.5-24.9** | **25.0-29.9** | **≥30.0** |
| **Unadjusted** | **Lowest** | 4.99 (4.33-5.76) [a] | 2.15 (1.87-2.47) [a] | 1.31 (0.94-1.82) | 2.44 (1.32-4.51) [b] |
| | **Middle** | 1.62 (1.30-2.03) [a] | Reference | 1.04 (0.76-1.42) | 0.89 (0.37-2.16) |
| | **Highest** | 0.24 (0.12-0.48) [a] | 0.42 (0.34-0.51) [a] | 0.48 (0.33-0.68) [a] | 0.70 (0.36-1.37) |
| **Model 1** | **Lowest** | 3.89 (3.36-4.49) [a] | 1.79 (1.56-2.06) [a] | 1.33 (0.96-1.85) | 3.47 (1.87-6.44) [a] |
| | **Middle** | 1.63 (1.31-2.05) [a] | Reference | 1.16 (0.85-1.59) | 1.46 (0.60-3.56) |
| | **Highest** | 0.36 (0.18-0.74) [b] | 0.57 (0.47-0.71) [a] | 0.72 (0.50-1.04) | 1.50 (0.77-2.94) |
| **Model 2** | **Lowest** | 3.92 (3.39-4.53) [a] | 1.80 (1.57-2.08) [a] | 1.34 (0.96-1.87) | 3.52 (1.89-6.54) [a] |
| | **Middle** | 1.65 (1.31-2.06) [a] | Reference | 1.17 (0.86-1.60) | 1.48 (0.61-3.63) |
| | **Highest** | 0.39 (0.19-0.79) [b] | 0.61 (0.50-0.75) [a] | 0.77 (0.54-1.11) | 1.65 (0.84-3.24) |
| **Model 3** | **Lowest** | 3.30 (2.83-3.85) [a] | 1.58 (1.37-1.83) [a] | 1.15 (0.82-1.61) | 2.82 (1.51-5.27) [b] |
| | **Middle** | 1.67 (1.33-2.10) [a] | Reference | 1.09 (0.80-1.48) | 1.23 (0.50-3.03) |
| | **Highest** | 0.41 (0.20-0.84) [c] | 0.60 (0.49-0.74) [a] | 0.70 (0.49-1.01) | 1.31 (0.66-2.57) |

**Cardiac death**

**Female**

| | | Categories of BMI (kg/m$^2$) | | | |
|---|---|---|---|---|---|
| | **Tertile of sCr** | **<18.5** | **18.5-24.9** | **25.0-29.9** | **≥30.0** |
| **Unadjusted** | **Lowest** | 4.58 (3.79-5.53) [a] | 2.44 (2.00-2.96) [a] | 1.42 (0.95-2.14) | 2.61 (1.36-5.01) [b] |
| | **Middle** | 0.87 (0.65-1.18) | Reference | 0.67 (0.40-1.10) | 1.26 (0.55-2.87) |
| | **Highest** | 0.26 (0.16-0.45) [a] | 0.33 (0.24-0.45) [a] | 0.45 (0.26-0.78) [b] | 0.14 (0.02-1.01) |
| **Model 1** | **Lowest** | 3.57 (2.95-4.33) [a] | 2.00 (1.64-2.44) [a] | 1.37 (0.91-2.06) | 3.05 (1.58-5.88) [b] |
| | **Middle** | 0.92 (0.68-1.24) | Reference | 0.71 (0.43-1.18) | 1.63 (0.71-3.72) |
| | **Highest** | 0.41 (0.24-0.71) [b] | 0.48 (0.35-0.66) [a] | 0.65 (0.38-1.13) | 0.25 (0.04-1.80) |
| **Model 2** | **Lowest** | 3.55 (2.93-4.30) [a] | 1.97 (1.61-2.41) [a] | 1.31 (0.87-1.98) | 2.89 (1.49-5.60) [b] |
| | **Middle** | 0.94 (0.70-1.27) | Reference | 0.69 (0.41-1.14) | 1.57 (0.69-3.60) |
| | **Highest** | 0.44 (0.26-0.76) [b] | 0.51 (0.37-0.70) [a] | 0.68 (0.39-1.18) | 0.26 (0.04-1.85) |
| **Model 3** | **Lowest** | 2.96 (2.42-3.60) [a] | 1.62 (1.32-1.98) [a] | 1.01 (0.66-1.53) | 2.00 (1.03-3.90) [c] |
| | **Middle** | 1.01 (0.75-1.36) | Reference | 0.59 (0.35-0.97) [c] | 1.23 (0.54-2.83) |
| | **Highest** | 0.49 (0.28-0.83) [b] | 0.50 (0.36-0.68) [a] | 0.57 (0.33-0.99) [c] | 0.18 (0.03-1.31) |

Data are expressed as odds ratio (95% confidence interval) compared to the reference group of BMI 18.5-24.9 with middle tertile of sCr.

Model 1: adjusted for age

Model 2: adjusted for age, dialysis vintage, diabetes mellitus

Model 3: adjusted for age, dialysis vintage, diabetes mellitus, serum albumin, phosphorus, C-reactive protein , Kt/V

[a] $p<0.001$, [b] $p<0.01$, [c] $p<0.05$ Abbreviation: BMI, body mass index; sCr, serum creatinine
