# Supplementary material for: Serum Creatinine Modifies Associations between Body Mass Index and Mortality and Morbidity in Prevalent Hemodialysis Patients
Source: PLoS One. 2016 Mar 1;11(3):e0150003. doi: 10.1371/journal.pone.0150003 (PMC4773191; doi:10.1371/journal.pone.0150003)
Supplement: S4 Table — (PDF) [file pone.0150003.s007.pdf]

S4 Table . Associations of BMI with cerebrovascular mortality according to Cr levels

Cerebrovascular death

Male

		**Categories of BMI (kg/m^2)**			
	**Tertile of sCr**	**<18.5**	**18.5-24.9**	**25.0-29.9**	**≥30.0**
**Unadjusted**	**Lowest**	3.18 (2.45-4.13) [a]	1.60 (1.25-2.05) [a]	1.40 (0.81-2.40)	1.91 (0.60-6.06)
	**Middle**	1.31 (0.87-1.98)	Reference	1.23 (0.76-2.01)	0.52 (0.07-3.73)
	**Highest**	0.35 (0.13-0.96) [c]	0.63 (0.46-0.85) [b]	0.64 (0.37-1.09)	0.69 (0.22-2.17)
**Model 1**	**Lowest**	2.96 (2.27-3.86) [a]	1.52 (1.18-1.95) [b]	1.41 (0.82-2.43)	2.12 (0.67-6.74)
	**Middle**	1.32 (0.87-1.99)	Reference	1.27 (0.78-2.08)	0.60 (0.08-4.31)
	**Highest**	0.40 (0.15-1.09)	0.69 (0.50-0.94) [c]	0.72 (0.42-1.24)	0.85 (0.27-2.70)
**Model 2**	**Lowest**	2.98 (2.28-3.88) [a]	1.52 (1.18-1.96) [b]	1.41 (0.81-2.43)	2.12 (0.67-6.74)
	**Middle**	1.33 (0.88-2.02)	Reference	1.27 (0.78-2.07)	0.60 (0.08-4.35)
	**Highest**	0.43 (0.16-1.18)	0.72 (0.53-0.99) [c]	0.75 (0.43-1.30)	0.90 (0.28-2.87)
**Model 3**	**Lowest**	2.58 (1.95-3.40) [a]	1.38 (1.07-1.79) [b]	1.28 (0.74-2.22)	1.81 (0.57-5.78)
	**Middle**	1.35 (0.89-2.03)	Reference	1.21 (0.74-1.98)	0.53 (0.07-3.85)
	**Highest**	0.46 (0.17-1.24)	0.72 (0.53-0.99) [c]	0.71 (0.41-1.24)	0.78 (0.24-2.50)

Cerebrovascular death

Female

		**Categories of BMI (kg/m^2)**			
	**Tertile of sCr**	**<18.5**	**18.5-24.9**	**25.0-29.9**	**≥30.0**
**Unadjusted**	**Lowest**	2.64 (1.88-3.70) [a]	1.90 (1.35-2.67) [a]	1.73 (0.92-3.26)	1.45 (0.35-5.99)
	**Middle**	1.13 (0.71-1.78)	Reference	0.34 (0.11-1.08)	2.40 (0.86-6.66)
	**Highest**	0.30 (0.13-0.71) [b]	0.48(0.30-0.75) [b]	0.28 (0.09-0.88) [c]	0.81 (0.20-3.34)
**Model 1**	**Lowest**	2.38 (1.68-3.36) [a]	1.75 (1.24-2.47) [b]	1.71 (0.91-3.20)	1.55 (0.37-6.39)
	**Middle**	1.15 (0.73-1.82)	Reference	0.34 (0.11-1.10)	2.67 (0.95-7.42)
	**Highest**	0.36 (0.16-0.85) [c]	0.56 (0.35-0.89) [c]	0.32 (0.10-1.03)	1.03 (0.25-4.28)
**Model 2**	**Lowest**	2.37 (1.67-3.34) [a]	1.70 (1.20-2.41) [b]	1.60 (0.84-3.02)	1.43 (0.34-5.92)
	**Middle**	1.19 (0.75-1.89)	Reference	0.33 (0.10-1.06)	2.52 (0.90-7.04)
	**Highest**	0.39 (0.17-0.92) [c]	0.58 (0.36-0.93) [c]	0.33 (0.10-1.05)	1.03 (0.25-4.29)
**Model 3**	**Lowest**	1.96 (1.37-2.79) [a]	1.46 (1.02-2.09) [c]	1.37 (0.72-2.60)	1.17 (0.28-4.87)
	**Middle**	1.23 (0.77-1.95)	Reference	0.30 (0.09-0.97) [c]	2.17 (0.77-6.07)
	**Highest**	0.42 (0.18-0.99) [c]	0.59(0.37-0.94) [c]	0.30 (0.09-0.97) [c]	0.87 (0.21-3.62)

Data are expressed as odds ratio (95% confidence interval) compared to the reference group of BMI 18.5-24.9 with middle tertile of sCr.

Model 1: adjusted for age

Model 2: adjusted for age, dialysis vintage, diabetes mellitus

Model 3: adjusted for age, dialysis vintage, diabetes mellitus, serum albumin, phosphorus, C-reactive protein , Kt/V

[a] $p<0.001$, [b] $p<0.01$, [c] $p<0.05$ Abbreviation: BMI, body mass index; sCr, serum creatinine
