# Supplementary material for: Serum Creatinine Modifies Associations between Body Mass Index and Mortality and Morbidity in Prevalent Hemodialysis Patients
Source: PLoS One. 2016 Mar 1;11(3):e0150003. doi: 10.1371/journal.pone.0150003 (PMC4773191; doi:10.1371/journal.pone.0150003)
Supplement: S5 Table — (PDF) [file pone.0150003.s008.pdf]

S5 Table. Associations of BMI with infection-related mortality according to Cr levels

Infection-related death

Male

	**Tertile of sCr**	**Categories of BMI (kg/m^2)** **<18.5**	**18.5-24.9**	**25.0-29.9**	**≥30.0**
**Unadjusted**	**Lowest**	8.08 (6.70-9.74) [a]	2.85 (2.36-3.44) [a]	1.25 (0.77-2.01)	0.00
	**Middle**	2.02 (1.51-2.72) [a]	Reference	0.59 (0.34-1.04)	0.00
	**Highest**	0.81 (0.46-1.43)	0.32 (0.24-0.45) [a]	0.21 (0.10-0.45) [a]	0.32 (0.08-1.30)
**Model 1**	**Lowest**	6.03 (4.98-7.30) [a]	2.30 (1.90-2.79) [a]	1.26 (0.78-2.04)	0.00
	**Middle**	2.03 (1.51-2.73) [a]	Reference	0.67 (0.38-1.19)	0.00
	**Highest**	1.31 (0.74-2.32)	0.47 (0.34-0.65) [a]	0.34 (0.16-0.73) [b]	0.78 (0.19-3.16)
**Model 2**	**Lowest**	6.11 (5.05-7.39) [a]	2.40 (1.98-2.91) [a]	1.37 (0.84-2.21)	0.00
	**Middle**	1.98 (1.47-2.66) [a]	Reference	0.70 (0.40-1.24)	0.00
	**Highest**	1.35 (0.76-2.39)	0.49 (0.35-0.68) [a]	0.37 (0.17-0.79) [c]	0.90 (0.2-3.66)
**Model 3**	**Lowest**	4.03 (3.30-4.93) [a]	1.85 (1.52-2.25) [a]	1.12 (0.69-1.83)	0.00
	**Middle**	1.92 (1.43-2.59) [a]	Reference	0.68 (0.39-1.21)	0.00
	**Highest**	1.50 (0.85-2.66)	0.53 (0.39-0.74) [a]	0.40 (0.19-0.85) [c]	0.89 (0.22-3.64)

Infection-related death

Female

	**Tertile of sCr**	**Categories of BMI (kg/m^2)** **<18.5**	**18.5-24.9**	**25.0-29.9**	**≥30.0**
**Unadjusted**	**Lowest**	7.92 (5.90-10.63) [a]	3.72 (2.74-5.06) [a]	2.71 (1.60-4.59) [a]	2.14 (0.67-6.91)
	**Middle**	1.11 (0.70-1.75)	Reference	1.32 (0.71-2.48)	1.76 (0.55-5.65)
	**Highest**	0.15 (0.05-0.48) [b]	0.23 (0.13-0.42) [a]	0.45 (0.18-1.13)	0.00
**Model 1**	**Lowest**	6.30 (4.67-8.48) [a]	3.11 (2.28-4.24) [a]	2.61 (1.54-4.43) [a]	2.46 (0.76-7.95)
	**Middle**	1.16 (0.73-1.83)	Reference	1.41 (0.75-2.64)	2.22 (0.69-7.17)
	**Highest**	0.22 (0.07-0.72) [c]	0.33 (0.18-0.60) [a]	0.64 (0.25-1.59)	0.00
**Model 2**	**Lowest**	6.26 (4.64-8.43) [a]	3.16 (2.32-4.32) [a]	2.67 (1.57-4.55) [a]	2.52 (0.78-8.18)
	**Middle**	1.15 (0.73-1.82)	Reference	1.42 (0.75-2.66)	2.29 (0.71-7.40)
	**Highest**	0.23 (0.07-0.74) [c]	0.35 (0.19-0.63) [a]	0.68 (0.27-1.71)	0.00
**Model 3**	**Lowest**	4.22 (3.11-5.73) [a]	2.31 (1.68-3.17) [a]	2.09 (1.22-3.58) [b]	1.88 (0.58-6.17)
	**Middle**	1.18 (0.74-1.87)	Reference	1.31 (0.69-2.46)	1.96 (0.60-6.34)
	**Highest**	0.26 (0.08-0.85) [c]	0.37 (0.20-0.67) [b]	0.67 (0.27-1.68)	0.00

Data are expressed as odds ratio (95% confidence interval) compared to the reference group of BMI 18.5-24.9 with middle tertile of sCr.

Model 1: adjusted for age

Model 2: adjusted for age, dialysis vintage, diabetes mellitus

Model 3: adjusted for age, dialysis vintage, diabetes mellitus, serum albumin, phosphorus, C-reactive protein , Kt/V

[a] $p<0.001$, [b] $p<0.01$, [c] $p<0.05$ Abbreviation: BMI, body mass index; sCr, serum creatinine
