# Supplementary material for: Serum Creatinine Modifies Associations between Body Mass Index and Mortality and Morbidity in Prevalent Hemodialysis Patients
Source: PLoS One. 2016 Mar 1;11(3):e0150003. doi: 10.1371/journal.pone.0150003 (PMC4773191; doi:10.1371/journal.pone.0150003)
Supplement: S6 Table — (PDF) [file pone.0150003.s009.pdf]

S6 Table. Associations of BMI with cancer mortality according to Cr levels

Cancer death

Male

		Categories of BMI (kg/m^2)			
	**Tertile of sCr**	**<18.5**	**18.5-24.9**	**25.0-29.9**	**≥30.0**
**Unadjusted**	**Lowest**	3.09 (2.45-3.91) [a]	2.17 (1.76-2.67) [a]	1.32 (0.81-2.16)	0.00
	**Middle**	1.21 (0.83-1.76)	Reference	0.97 (0.60-1.56)	0.41 (0.06-2.92)
	**Highest**	0.62 (0.32-1.23)	0.25 (0.17-0.36) [a]	0.27 (0.13-0.54) [a]	0.18 (0.03-1.29)
**Model 1**	**Lowest**	2.48 (1.96-3.15) [a]	1.86 (1.50-2.29) [a]	1.34 (0.82-2.19)	0.00
	**Middle**	1.22 (0.83-1.77)	Reference	1.07 (0.66-1.72)	0.62 (0.09-4.48)
	**Highest**	0.90 (0.46-1.78)	0.32 (0.22-0.47) [a]	0.38 (0.19-0.78) [b]	0.34 (0.05-2.47)
**Model 2**	**Lowest**	2.51 (1.98-3.19) [a]	1.94 (1.56-2.40) [a]	1.45 (0.88-2.38)	0.00
	**Middle**	1.17 (0.80-1.71)	Reference	1.11 (0.68-1.79)	0.66 (0.09-4.78)
	**Highest**	0.85 (0.43-1.68)	0.31 (0.21-0.46) [a]	0.39 (0.19-0.79) [b]	0.35 (0.05-2.55)
**Model 3**	**Lowest**	1.51 (1.17-1.94) [b]	1.39 (1.12-1.73) [b]	1.11 (0.67-1.83)	0.00
	**Middle**	1.16 (0.79-1.69)	Reference	1.08 (0.67-1.75)	0.61 (0.09-4.41)
	**Highest**	0.99 (0.50-1.96)	0.36 (0.24-0.52) [a]	0.43 (0.21-0.89) [c]	0.38 (0.05-2.76)

Cancer death

Female

		Categories of BMI (kg/m^2)			
	**Tertile of sCr**	**<18.5**	**18.5-24.9**	**25.0-29.9**	**≥30.0**
**Unadjusted**	**Lowest**	3.02 (2.01-4.54) [a]	2.52 (1.69-3.76) [a]	1.53 (0.68-3.45)	0.00
	**Middle**	1.16 (0.66-2.03)	Reference	0.85 (0.33-2.17)	2.72 (0.83-8.88)
	**Highest**	0.46 (0.19-1.09)	0.46 (0.26-0.82) [b]	0.42 (0.13-1.36)	0.61 (0.08-4.49)
**Model 1**	**Lowest**	2.50 (1.65-3.78) [a]	2.17 (1.45-3.25) [a]	1.48 (0.66-3.34)	0.00
	**Middle**	1.20 (0.69-2.11)	Reference	0.89 (0.35-2.28)	3.29 (1.00-10.80) [c]
	**Highest**	0.64 (0.27-1.54)	0.62 (0.35-1.10)	0.55 (0.17-1.81)	0.95 (0.13-6.99)
**Model 2**	**Lowest**	2.51 (1.66-3.80) [a]	2.24 (1.49-3.37) [a]	1.57 (0.69-3.57)	0.00
	**Middle**	1.17 (0.67-2.06)	Reference	0.92 (0.36-2.36)	3.47 (1.05-11.43) [c]
	**Highest**	0.62 (0.26-1.50)	0.61 (0.34-1.09)	0.56 (0.17-1.84)	0.99 (0.13-7.26)
**Model 3**	**Lowest**	1.72 (1.12-2.64) [c]	1.67 (1.10-2.54) [c]	1.22 (0.53-2.81)	0.00
	**Middle**	1.18 (0.67-2.09)	Reference	0.85 (0.33-2.18)	3.00 (0.91-9.92)
	**Highest**	0.71 (0.29-1.71)	0.65 (0.36-1.16)	0.55 (0.17-1.81)	0.90 (0.12-6.63)

Data are expressed as odds ratio (95% confidence interval) compared to the reference group of BMI 18.5-24.9 with middle tertile of sCr.

Model 1: adjusted for age

Model 2: adjusted for age, dialysis vintage, diabetes mellitus

Model 3: adjusted for age, dialysis vintage, diabetes mellitus, serum albumin, phosphorus, C-reactive protein , Kt/V

[a] $p<0.001$, [b] $p<0.01$, [c] $p<0.05$ Abbreviation: BMI, body mass index; sCr, serum creatinine
